# Molecular and clinical features of papillary thyroid cancer in adult patients with a non-classical phenotype

**DOI:** 10.3389/fendo.2023.1138100

**Published:** 2023-04-12

**Authors:** Jie Zhou, Wei-Ran Wang, Hui-Fang Zhang, Qi-Qi Gao, Wei-Bin Wang, Jian-Hua Zhu, Yu-Shuai Han, Jing Chen, Tong-Hui Ma, Xiao-Yan Zhang, Xiao-Dong Teng

**Affiliations:** ^1^ Department of Pathology, First Affiliated Hospital, Zhejiang University School of Medicine, Hangzhou, China; ^2^ Department of Translational Medicine, Genetron Health (Beijing) Technology, Co. Ltd., Beijing, China; ^3^ Cancer Center, First Affiliated Hospital, Zhejiang University School of Medicine, Hangzhou, China

**Keywords:** papillary thyroid carcinoma, phenotype, gene fusion, ETV6-NTRK3, NCOA4-RET

## Abstract

**Purpose:**

Genotyping is fundamental in papillary thyroid cancer (PTC) and helps to enhance diagnosis and prognosis and determine appropriate treatments. The phenotype-genotype association in PTC was previously studied, with *BRAF* V600E characterizing classic PTC and tall-cell PTC and *RAS* mutations characterizing follicular-variant PTC. In clinic, some non-classical histological subtypes of PTC were also identified, however, their genotype remains unclear. In this study, we collected samples of these non-classical PTC after the exclusion of classic phenotypes and examined their phenotypes, genotype and the relationship between phenotype and genotype.

**Methods:**

We screened out non-classical PTC by excluding classical PTC from 1,059 different thyroid samples, and a total of 24 cases was obtained and described from the morphological features, which is rare in differentiated PTC. DNA/RNA sequencing was performed using 18 available samples to describe the genetic features.

**Results:**

PTC with the non-classical phenotype were characterized cuboidal to low columnar tumor cells with subtle nuclear features of PTC and without discernible nuclear elongation, concurrently with dense microfollicles, delicate papillae or solid nodules with delicate fibrovascular cores. They were associated with lymphatic vessel invasion (P<0.001) but not with a worse prognosis (P=0.791). Gene fusions were identified in 14 of 18 (77.8%) cases, including eight fusions of *NTRK* and six fusions of *RET*. The high percentage of fusions in this papillary thyroid cancer subgroup suggested a correlation of gene fusions with the phenotype that does not belong to the *BRAF* V600E-mutant or *RAS*-mutant group.

**Conclusions:**

Our study retrospectively screened a large cohort of different thyroid tissue samples, and presented the histopathological and genetic features of a non-classical phenotype of PTC from 24 patients. It may contribute to diagnose in PTC, and patients of these non-classical phenotype may benefit from targeted therapy, compared to a natural patient cohort without selection.

## 1 Introduction

Globally, the incidence of thyroid cancer has markedly increased in the past 30 years (1, 2), and the prevalence of diverse histological and genetic characteristics has changed over time (3). With the exception of medullary thyroid cancer, all thyroid cancers are derived from follicular cells. About 80% to 85% of all thyroid cancers are papillary thyroid cancers (PTCs) (4–6), named for their papillary histological architecture. PTCs include several subtypes, such as classic PTC (CPTC), follicular-variant PTC (FVPTC), tall-cell PTC (TCPTC), and a few other rare variants (7, 8).

*BRAF* V600E and *RAS* mutations have been identified as the primary genetic drivers in thyroid carcinomas (9–11), followed by fusions involving *RET (* 12) and other receptor tyrosine kinases (13). It is worthwhile pointing out that these mutations are almost always mutually exclusive (14). The *BRAF* V600E mutation is the most frequent genetic change in PTC, with a prevalence between 30 to 80% (15–17), and appears to be associated with a higher risk of cancer recurrence (18). The *RAS* oncogene family includes *HRAS*, *NRAS*, and *KRAS*. The prevalence of *RAS* mutations in thyroid cancer is approximately 20–40%, and the mutations are found in follicular lesions, including adenomas, carcinomas, and FVPTCs (19). Differentiated thyroid tumors harboring *RAS* mutations without co-alterations have an excellent prognosis (20). The Cancer Genome Atlas (TCGA) described a genomic landscape of 496 PTCs and confirmed the phenotype-genotype association in PTCs, with *BRAF* V600E characterizing CPTC and TCPTC and *RAS* mutations characterizing FVPTC (21).

*RET* fusion can result in oncogenic activation and occurs in about 5–35% of PTCs (22–24), whereas the rearrangements of other *RTKs* such as *NTRK1/3, ALK*, and *ROS1* translocations have been reported in a minor subset of PTCs (25–31). The associations of gene fusions with the outcome of the disease are not yet well understood. Yuri E Nikiforov’s group described the morphological features of *ETV6-NTRK3*-positive PTC in adults without radiation exposure with the tumors exhibiting an admixture of papillary and follicular areas, clear cell or oncocytic foci with overt nuclear features of PTC, interspersed bland areas, glomeruloid follicles, reversed nuclear polarization and cytoplasmic vacuolization (32). However, this study did not propose a clear morphological standard for *NTRK* fusion-driven PTCs. At the same time, there are PTCs with histological characteristics that are non-classical from known pathological subtypes, and their genotypes are unclear. Thus, the relationship between morphological characteristics and genotypes still warrants further investigation.

Herein, 1,059 patients with thyroid cancer, who underwent surgery from 2008 to 2015, were retrospectively analyzed. A total of 24 PTC cases were identified according to morphological features that are different from the known subtypes. Molecular analyses were performed and revealed a high prevalence of gene fusion, while no mutations in *BRAF, RAS*, or *TERT* genes were detected. Identification of the relationship between these cytological morphologies and genomic fusions or clinical features will facilitate the rationalization of clinical treatment strategies.

## 2 Methods

### 2.1 Patients and samples

The study was conducted according to the guidelines of the Declaration of Helsinki, and approved by the Ethics Committee of the First Affiliated Hospital College of Medicine, Zhejiang University (NO. IIT20210692A). As a retrospective research, the data are anonymous, the requirement for informed consent was therefore waived.

A total of 1,059 thyroid cancer tissues were collected from patients who underwent thyroid surgery at The First Affiliated Hospital, Zhejiang University School of Medicine (Hangzhou, China) between 2008 and 2015. All available samples were fresh frozen thyroid tissues. Detailed clinical and pathological data were collected from patients harboring primary dominant PTCs with dimensions ≥1 cm.

### 2.2 PCR and sequencing of *BRAF* V600E

The genomic DNA was isolated using a commercial kit (QIAamp DNA Mini Kit; QIAGEN, Hilden, Germany). Exon 15 of the *BRAF* gene was amplified and then directly sequenced for *BRAF* V600E mutation as described previously (33).

### 2.3 Next-generation sequencing analysis

#### 2.3.1 Thyroid NGS panel sequencing

The DNA and RNA extracted from PTC samples with the non-classical phenotype were analyzed with the Onco-Thyroid™ panel (Genetron Health, Beijing, China, gene list is in **Table S1**) using one-step multiplex PCR targeted amplicons, as previously described (34).

#### 2.3.2 RNA panel sequencing

Fusioncapture™ (GenetronHealth), which is a 395-gene RNA panel (gene list is in **Table S2**), was conducted to identify gene fusions in the PTC samples with the non-classical phenotype. The specific method is stated in the previous paper (35).

#### 2.3.3 Whole-exome sequencing

Genomic DNA from PTC samples with the non-classical phenotype was fragmented for constructing a library using KAPA Hyper Prep kits (KAPA, KK8504) and captured using the Agilent SureSelect XT Human All Exon v5 kit (Agilent, Santa Clara, CA, USA). High-throughput sequencing was performed as previously described (36).

#### 2.3.4 Statistical analyses

Comparisons of categorical variables were performed by Pearson’s chi-squared test. The analyses and data presentation were carried out using GraphPad Prism (8.0.1).

## 3 Results

### 3.1 Sample selection

**Figure 1:**
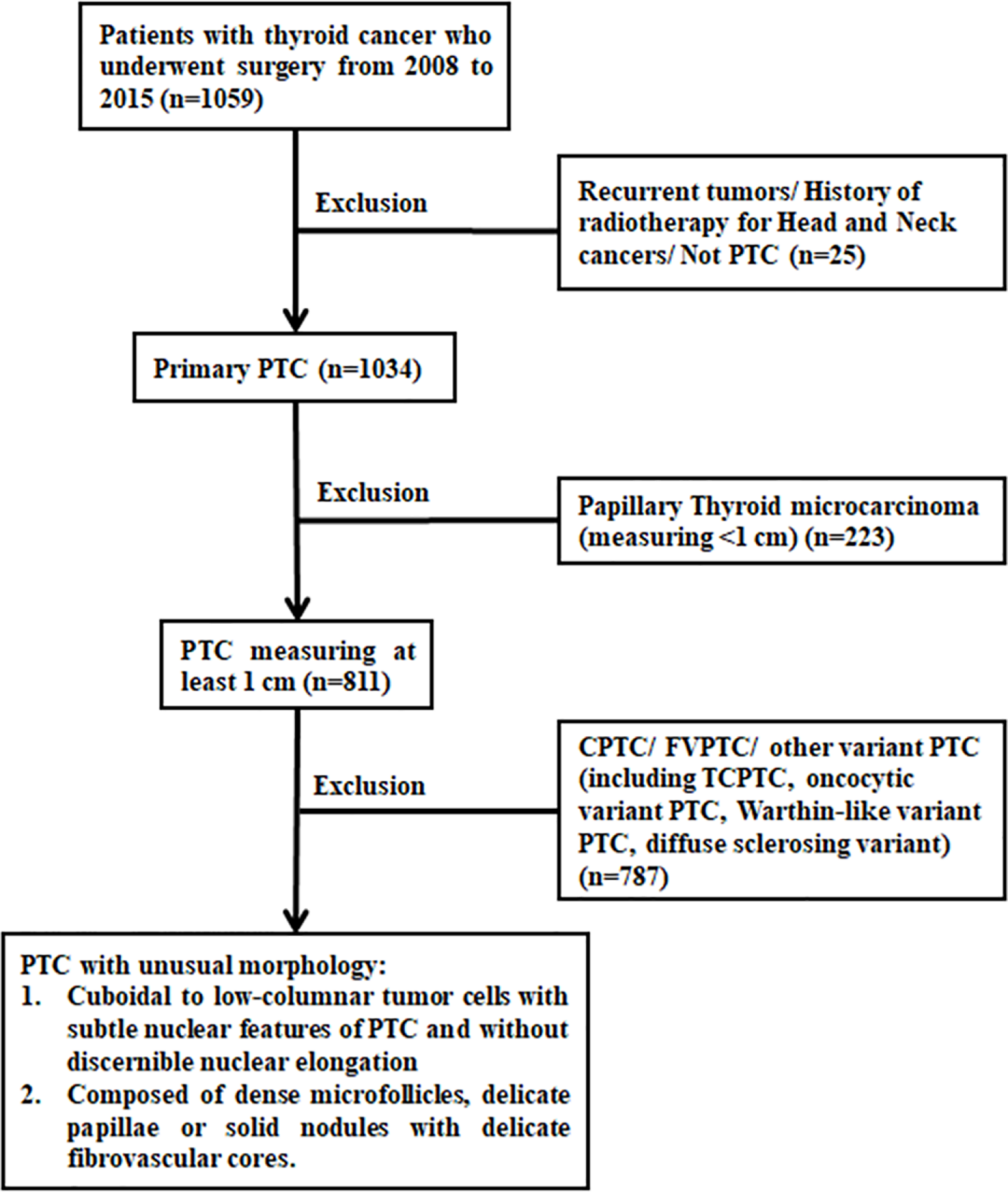
Workflow of the study sample selection.

**Figure 1** illustrates the study sample selection workflow. Of the 1,059 patients who underwent thyroid surgery at The First Affiliated Hospital, Zhejiang University School of Medicine (Hangzhou, China) between 2008 and 2015, 25 patients whose tumors were not primary differentiated PTC were first excluded, then 223 patients with papillary thyroid microcarcinoma were excluded. Finally, 787 PTC patients with common histopathologies, such as CPTC, FVPTC, TCPTC, oncocytic variant of PTC, the Warthin-like variant of PTC, and the diffuse sclerosing variant of PTC, were excluded. The final study sample consisted of 24 adult participants with no prior history of radiation exposure with a phenotype non-classical from common PTCs. The tumors were characterized as the following criteria: 1. Cuboidal to low-columnar tumor cells with subtle nuclear features of PTC and without discernible nuclear elongation. 2. Composed of dense microfollicles, delicate papillae or solid nodules with delicate fibrovascular cores.

### 3.2 Clinical characteristics

The clinical and pathologic features of PTC patients with the non-classical phenotype are summarized (**Table 1**, **Table S3**). The prevalence of PTC samples measuring at least 1 cm was 3.0% (24/811). There were 19 (79.2%) females and 5 (20.8%) males. The age at initial diagnosis ranged from 19 to 70 years (mean 42.1 years). The tumor size ranged from 1.0 cm to 7.0 cm (mean 1.8 cm). Moreover, the tumors were often described as solitary nodules (87.5%). Additionally, 416 PTC patients with normal phenotypes and clinical information were also included in the analysis (**Table 1**, **Table S4**). Overall, the non-classical phenotype was significantly associated with lymphatic vessel invasion, a strong prognostic factor for poor clinical outcomes in lymph node-negative patients with breast carcinoma, bladder cancer, non-small cell lung cancer, colorectal cancer and differentiated thyroid carcinoma (37–42). While tumor recurrence was 6.3% (26 of 416) in patients with a normal phenotype, it was 0% (0 of 24) in patients with the non-classical phenotype (P = 0.791).

**Table 1:** Relationship of the non-classical phenotype group with clinicopathologic outcomes.

Clinicopathologic Outcomes	Normal Phenotype (n = 416)	Non-classical Phenotype (n = 24)	P
	No. (%)	No. (%)	
Age at diagnosis, years, Means ± SD	44.4 ± 12.6	42.1 ± 13.4	0.388
Sex, female	298 (71.6)	19 (79.1)	0.424
Size, cm, Means ± SD	1.7 ± 0.9	1.8 ± 1.2	0.483
Lymph node metastasis	195 (46.9)	14 (58.3)	0.274
Multifocality	126 (30.3)	3 (12.5)	0.063
Lymphatic vessel invasion	38 (9.1)	14 (58.3)	<0.001
Extrathyroidal Extension	83 (20.0)	6 (25)	0.736
Tumor recurrence	26 (6.3)	0 (0)	0.414
Total follow-up, months, Median (IQR)	69 (59-84)	86 (18-127)	0.791

IQR, interquartile range; SD, standard deviations.

### 3.3 Histopathological features of PTC with the non-classical phenotype

**Figure 2:**
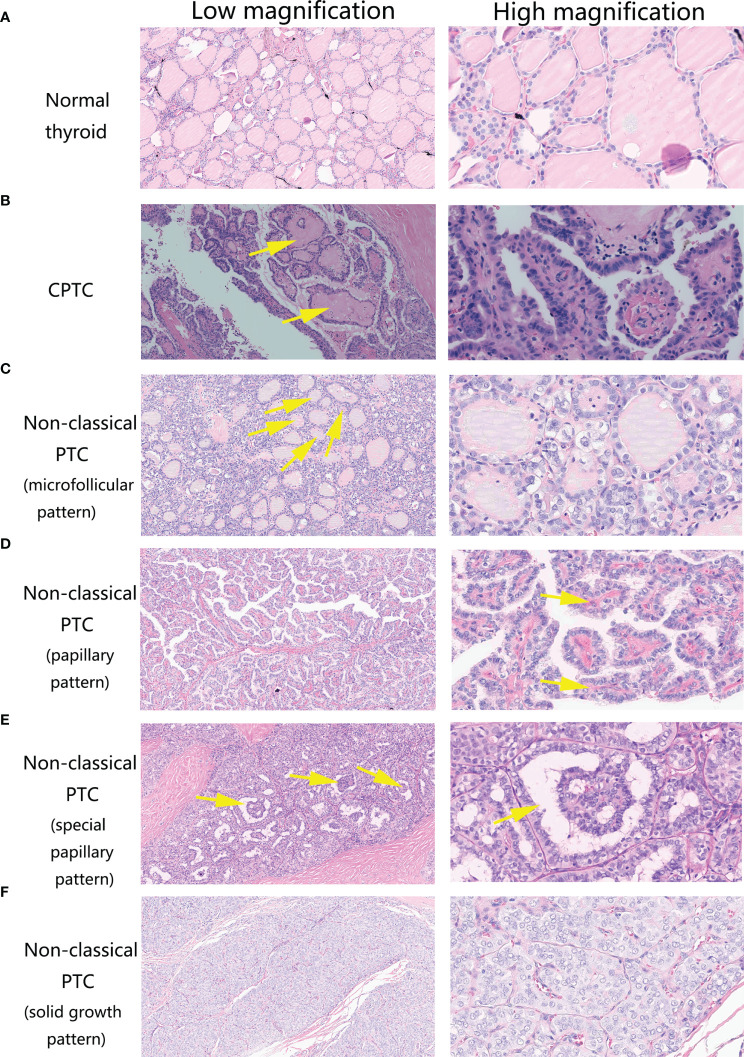
Growth patterns of Papillary Thyroid Carcinomas tumors with non-classical phenotype and controls in 100X and 400X magnification. **(A)** Normal thyroid. **(B)** Classic PTC: low magnification shows the papillae found in CPTC usually vary in size and shape and some have broader central stalks. The arrow shows the papillae with broader central stalks. High magnification shows that the papillae composed of columnar epithelial cells. The nucleus has classic characteristics nuclear features of PTC and with significant elongation. **(C)** Dense microfollicles are composed of small cuboidal epithelia with round nuclei intermixed with some variable shaped nuclei. The tumor cells have transparent or pale cytoplasm with ill-defined borders. Arrows show the microfollicles. **(D)** Tumor is dominantly composed of delicate papillae (arrow). **(E)** Occasionally tumors present glomeruloid bodies resembling abortive papillae. **(F)** Solid growth pattern, characterized by delicate fibrovascular cores within the solid nodules. The tumor cells have nucleus without significant elongation.

**Figure 3:**
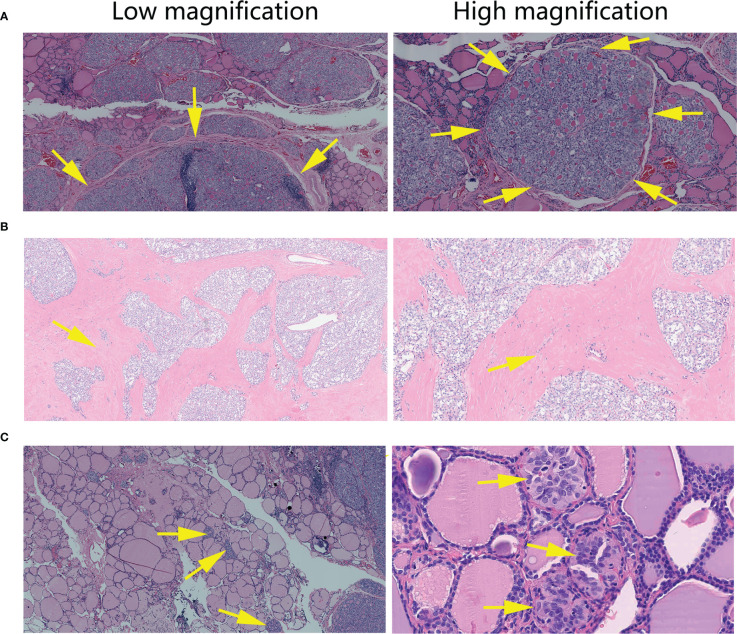
Histopathological features of Papillary Thyroid Carcinomas tumors with non-classical phenotype. **(A)** In 40X and 100X magnification, tumor tissue arranged in smooth-bordered nodules (arrow), as separated by normal thyroid tissue. **(B)** In 40X and 100X magnification, tumor tissue arranged in a geographic, jigsaw-like pattern within a broad fibrous background (arrow). **(C)** 40X and 400X magnification shows small satellite nodules (arrow) near or away from the dominant tumor.

Microscopic examination revealed that all 24 tumors were lack of normal follicular structure of thyroid tissue and normal cell with small round-flat nucleus (**Figure 2A**). They were uniformly composed of small cuboidal to low columnar epithelial cells. The nucleus had subtle characteristics nuclear features of PTC and without significant elongation, in contrast, CPTC composed of columnar epithelial cells and the nucleus had classic characteristics nuclear features of PTC and with significant elongation (**Figure 2B**). Some nuclei were pushed by the cytoplasm and formed variable shapes such as rings and triangles. Furthermore, the cytoplasm was transparent or pale, and the borders of tumor cells were ill-defined. The tumors with non-classical phenotype showed three main patterns of growth. Firstly, it is the microfollicular pattern, which characterized by dense microfollicles (**Figure 2C**). The second one is papillary pattern, which characterized by monotonous delicate papillae with a fine central fibrovascular stalk (**Figure 2D**), of which the delicate papillae are quite different from those in CPTC (**Figure 2B**). Occasionally, tumors presented glomeruloid bodies resembling abortive papillae (**Figure 2E**). The last pattern is solid growth, characterized by delicate fibrovascular cores within the solid nodules or islands (**Figure 2F**). Tumors with the non-classical phenotype were mostly poorly circumscribed and arranged in pushing, smooth-bordered nodules or islands of variable sizes, as separated by normal thyroid tissue (**Figure 3A**) or broad fibrous septa (jigsaw-like pattern) (**Figure 3B**). Often, there were small satellite nodules near or away from the dominant tumor (**Figure 3C**). Conventional aggressive features of PTC, including extrathyroidal extension, lymphatic vessel invasion, and lymph node metastases, were present in 6/24, 14/24, and 14/24 cases, respectively (**Table 1**, **Table S3**).

### 3.4 Molecular findings of PTC with the non-classical phenotype

**Figure 4:**
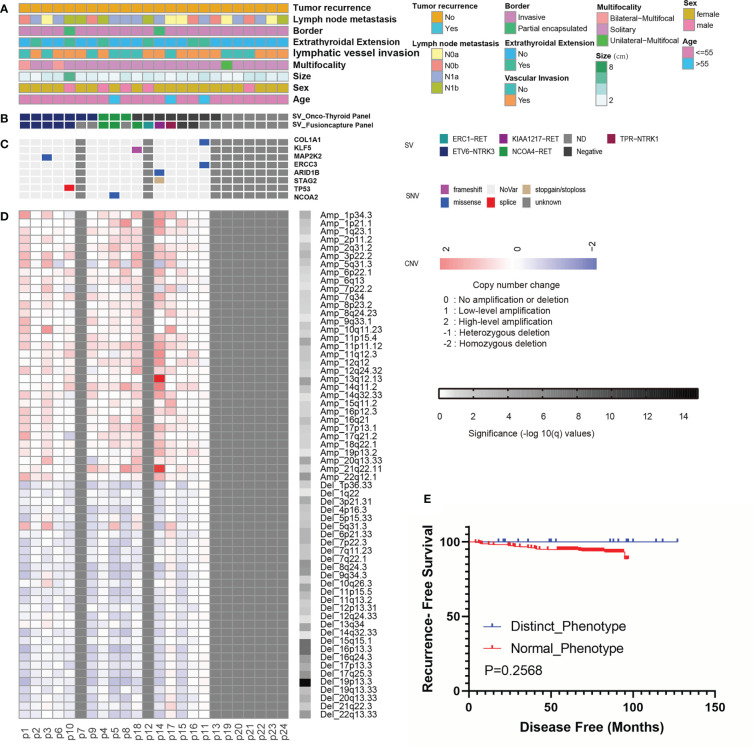
Landscape of genomic alterations and Kaplan-Meier analyses in Papillary Thyroid Carcinomas with non-classical phenotype. **(A-D)** Landscape of genomic alterations (n=24). **(A)** Tumor recurrence, lymph node metastasis, border, extrathyroidal extension, lymphatic vessel invasion, multifocality, tumor size, patient gender and age. **(B)** Fusion events detected by Onco-Thyroid Panel and Fusioncapture Panel sequencing. **(C)** Mutations of driver genes detected by WES. **(D)** Copy number changes detected by WES. **(E)** Kaplan-Meier analyses of the impacts of phenotype on recurrence-free survival of PTC patients (n=18).

*BRAF* V600E was analyzed using ARMS-PCR (AmoyDx, China) in all the tumors with the non-classical phenotype (n = 24), and molecular analyses revealed non-BRAF V600E. Meanwhile, the percentage of BRAF V600E positive patients in our control cohort was 72.6% (302/416). For the cases with remaining tumor samples (n = 18), Onco-thyroid panel (18/18), RNA panel (13/18), and whole-exome (15/18) sequencing were performed (**Figures 4A–D**, **Tables S5**–**S7**) to acquire additional genetic information. Fourteen tumors (77.8%, 14/18) harboring gene fusions were recognized by the Onco-thyroid panel and/or Fusioncapture sequencing, seven of which were ETV6-NTRK3, one was TPR-NTRK1, and the others were fusions of the RET gene (**Figures S1**, **S2**, **Table S3**). *RET* fusions were most frequent (33/484, 6.8%) in the TCGA study, and the most common partner gene was *CCDC6* (21/33, 63.6%) (21). Meanwhile, *NCOA4-RET* was recognized in four of cases (4/6, 66.7%) with the non-classical phenotype in this study and no *CCDC6-RET* was identified. More gene mutations, copy number variation (CNV), and tumor mutation burden (TMB) were detected and calculated by whole-exome sequencing, and the results showed that there were no other well-known driver mutations of PTC (**Table S6**). Notably, driver variations were not detected in 4 of the 13 patients, which indicates that the molecular mechanism of thyroid cancer still warrants further investigation. Extensive fibrosis was commonly encountered in our series, and cases with *RET* fusion (6/6) were more prone to severe fibrosis than those with the *NTRK* fusion (1/8). Besides, more patients with *NTRK* fusions (4/8) in the group appeared to have a high level of thyroid peroxidase antibody (TPO) compared to patients with *RET* fusions (1/6) (**Table S3**). However, the result of Fisher’s exact test suggested no statistically difference in TPO levels (P=0.3007), which may due to small sample size.

### 3.5 Recurrence-free survival in patients with the non-classical phenotype

Follow-up data were available for 18 patients (median 86 months, range 18-127 months): all patients were alive and without recurrence (**Table S3**). The effects of non-classical and normal phenotypes on PTC recurrence were compared. Although non-classical phenotypic tumors were more likely to have lymphatic vessel invasion (**Table 1**), they did not show an elevated risk for recrudescence (**Figure 4E**).

## 4 Discussion

Key genetic alterations of PTC, co-occurring or mutually exclusive, are the basis of estimated prognosis. The phenotype-genotype association in PTCs was described as *BRAF* V600E characterizing CPTC, TCPTC and *RAS* mutations characterizing FVPTC (21). Numerous studies have demonstrated that co-occurrence of *BRAF* V600E and *TERT* promoter mutations defines an aggressive subgroup of PTCs (43–49). On the one hand, differentiated thyroid cancers harboring *RAS* mutations alone have an excellent prognosis (20). On the other hand, PTCs with a novel phenotype that do not belong to the *BRAF* V600E-mutant or *RAS*-mutant tumors should be better characterized, given that some of these tumors harbor gene fusions that can benefit from targeted therapy. As FDA approves larotrectinib and entrectinib for solid tumors with *NTRK* gene fusions, and pralsetinib and selpercatinib for thyroid cancers with *RET* gene mutations or fusions.

Herein, we retrospectively analyzed a large cohort of thyroid tissue samples, screened out well-differentiated PTCs, and excluding PTMC, CPTC, FVPTC, TCPTC, oncocytic variants of PTC, the Warthin-like variant of PTC, and the diffuse sclerosing variant of PTC. A non-classical phenotype of PTC was summarized from 24 patients. The microfollicular architecture consisted of closely-packed small follicles with scanty colloids, different from the common FVPTC. The papillary structure could be predominant or even exclusive, but it was always monotonous, delicate. In comparison, the papillae found in CPTC usually vary in size and shape and generally have broader central stalks. We presumed that these delicate papillae were formed in the following ways: (1) originating from a solid structure with delicate fibrovascular cores by cellular dehiscence (**Figure S3**); or (2) originating from packed microfollicles by follicular-septum cracking, which may be different from the formation of the common papillae present in CPTC (**Figure S4**). Meanwhile, considering that the solid growth pattern was predominant (> 50%) in our cases, a diagnosis of a solid variant of PTC (SVPTC) may also be established (50, 51), which is most commonly seen in children after the Chernobyl nuclear accident (52, 53) or in few adults without a history of radiation exposure (51), accounting for 1-3% of all PTCs (54). Different from the most common encapsulated type (65%) (54) in reported SVPTC, our general solid growth cases were almost non-encapsulated and invasive (87.5%, 7/8), and showed a lower proportion (0.76%, 8/1059) in Chinese, suggest it may be clinically rare. Furthermore, extensive fibrosis was quite common in our series. Although sometimes seen in CPTC, the tumor nests were more irregular and infiltrative. Other features, including clear or pale cytoplasm, no elongated nuclei, and smaller and shorter cells, are also of tremendous significance in these subtypes of PTC.

We selected 416 patients with a common PTC phenotype who underwent surgery in the hospital from 2008 to 2015 as a real-world data control to explore whether PTC patients with a non-classical phenotype have significant differences in clinical characteristics. The median age of PTC patients with the non-classical phenotype (42 years old) was similar to that of PTC patients with a common phenotype (44 years old) (P = 0.388). The female to male ratios were 2.5:1 and 3.8:1 in the common and non-classical phenotype of PTC groups (P = 0.424), respectively. For aggressive features such as invasive border, extrathyroidal extension, lymphatic vessel invasion, and lymph node metastases, PTCs with the non-classical phenotype showed a significant difference in lymphatic vessel invasion (P < 0.001), whereas there was no difference in tumor recurrence (P = 0.414). The results suggest that although there are differences in histopathological features between common and non-classical phenotypes of PTC, clinical characteristics and recurrence are relatively similar. Among the 24 cases of PTC with a non-classical phenotype, 18 had fresh frozen tissues stored. After Onco-Thyroid and/or RNA panel sequencing, 14 gene fusions were recognized, including 8 fusions in *NTRK* and 6 fusions in *RET*. In the thyroid, *NTRK*-driven malignancies are rare, and their frequency ranges from 2 to 3% in adult patients without radiation exposure (21, 55–57). However, the frequency of *NTRK* fusion-positive PTCs was 44.4% (8/18) in this group. *RET* fusions occur in 5%–10% of sporadic PTCs (58, 59). In the TCGA study, 6.8% of cases harbored a *RET* fusion (21). In contrast, *RET* fusions were observed in 33.3% (6/18) of PTCs with the non-classical phenotype in this study. The results indicate that the fusions of *NTRK* and *RET* are associated with the non-classical phenotype of PTC. Meanwhile, in this phenotype of PTC, cases with *RET* fusion are more prone to fibrosis than those with *NTRK* fusion. Various *RET* and *NTRK* fusions have been reported in PTC. The two most prevalent *RET* fusions are *CCDC6-RET* and *NCOA4-RET*, which account for more than 90% of all *RET* rearrangements (60–62). *NTRK* fusions discovered in thyroid tumors so far include *EML4-NTRK3* (57), *ETV6-NTRK3* (26, 27, 63), *SQSTM1-NTRK3* (57), *IRF2BP2-NTRK1* (57), *TPR-NTRK1* (26, 27), *TPM3-NTRK1* (25, 64), *TFG-NTRK1* (64, 65), and *TRIM33-NTRK1* (65). Interestingly, the common fusions of *RET/NTRK3* discovered in this study are *NCOA4-RET* (4/6) and *ETV6-NTRK3* (7/7), showing selectivity of fusion partners. Meanwhile, except for one mutation in *TP53*, no mutations leading to the occurrence of PTC were found in other common genes (such as *AKT1, BRAF, CTNNB1, EIF1AX, EZH1, GNAS, HRAS, KRAS, NRAS, PIK3CA, RET, SPOP, TERT, TSHR, ZNF148*, and so on), signaling the pivotal role of gene fusion in this group of PTC. Recently, there has been a growing interest in testing and characterizing *NTRK* and *RET* fusion genes because they are therapeutically targetable. For instance, *NTRK* and *RET* fusion-positive tumors are sensitive to inhibitors, such as larotrectinib and selpercatinib, which appear to be well tolerated and effective (58, 66, 67). Due to the limited sample size in current research, it is not yet possible to have an impact on clinical treatment options for those patients. However, our study provides a possibility that this type of non-classical PTC may be classified together to spare these patients unnecessary aggressive therapy, as they shared similar molecular and clinical features and had a relatively good prognosis.

## 5 Conclusion

In conclusion, our study retrospectively screened a large cohort of different thyroid tissue samples, and a non-classical phenotype of PTC was summarized from 24 patients. Herein, the histopathological and genetic features of this subgroup of PTC were presented. Our findings may facilitate diagnostic approaches in PTC, and patients of this peculiar subgroup may benefit from targeted therapy compared to a natural patient cohort without selection. However, this study was based on a small population, and genetic testing were not performed on all tissue samples. In the future, further analyses are warranted to corroborate the results.

## Data availability statement

The raw datasets presented in this study can be found in the National Omics Data Encyclopedia (NODE) database (https://www.biosino.org/node/index), accession number OEP003392. 

## Ethics statement

The studies involving human participants were reviewed and approved by the Ethics Committee of the First Affiliated Hospital College of Medicine, Zhejiang University (NO. IIT20210692A). Written informed consent for participation was not required for this study in accordance with the national legislation and the institutional requirements.

## Author contributions

Conceptualization, JZ and X-DT. Methodology, JZ, W-RW, T-HM and X-YZ. Software, H-FZ, Q-QG, and X-YZ. Validation, JZ, W-RW, W-BW, J-HZ and Y-SH. Formal Analysis, JZ, W-RW, H-FZ, Q-QG, W-BW, Y-SH, T-HM, X-YZ and X-DT. Investigation, JZ and W-RW. Resources, X-DT. Data Curation, JZ and W-RW. Writing – Original Draft Preparation, JZ, W-RW. Writing – Review & Editing, JC, T-HM and XDT. Visualization, JZ and W-RW. Funding – T-HM. Supervision, X-DT. Project Administration, X-DT. All authors contributed to the article and approved the submitted version.

## Supplementary material

Supplementary Figure 1 The Integrative Genomics Viewer (IGV) screenshots display the reads from next-generation sequencing by Onco-Thyroid panel and reveal harbouring of gene fusions. The reference is fusion gene sequences.

Supplementary Figure 2 The IGV screenshots display the reads from next-generation sequencing by Fusioncapture panel and reveal harbouring of gene fusions. The reference is GRCh37/hg19.

Supplementary Figure 3 The delicate papillae result from cellular dehiscence, with rugged surface and adhesion.

Supplementary Figure 4 Putative scheme of papillae formations. **(A)** The delicate papillae originate from packed microfollicles. **(B)** The common papillae originate from irregular follicles.
