# Supplementary figures and images for: Molecular and clinical features of papillary thyroid cancer in adult patients with a non-classical phenotype
Source: Front Endocrinol (Lausanne). 2023 Apr 12;14:1138100. doi: 10.3389/fendo.2023.1138100 (PMC10130378; doi:10.3389/fendo.2023.1138100)

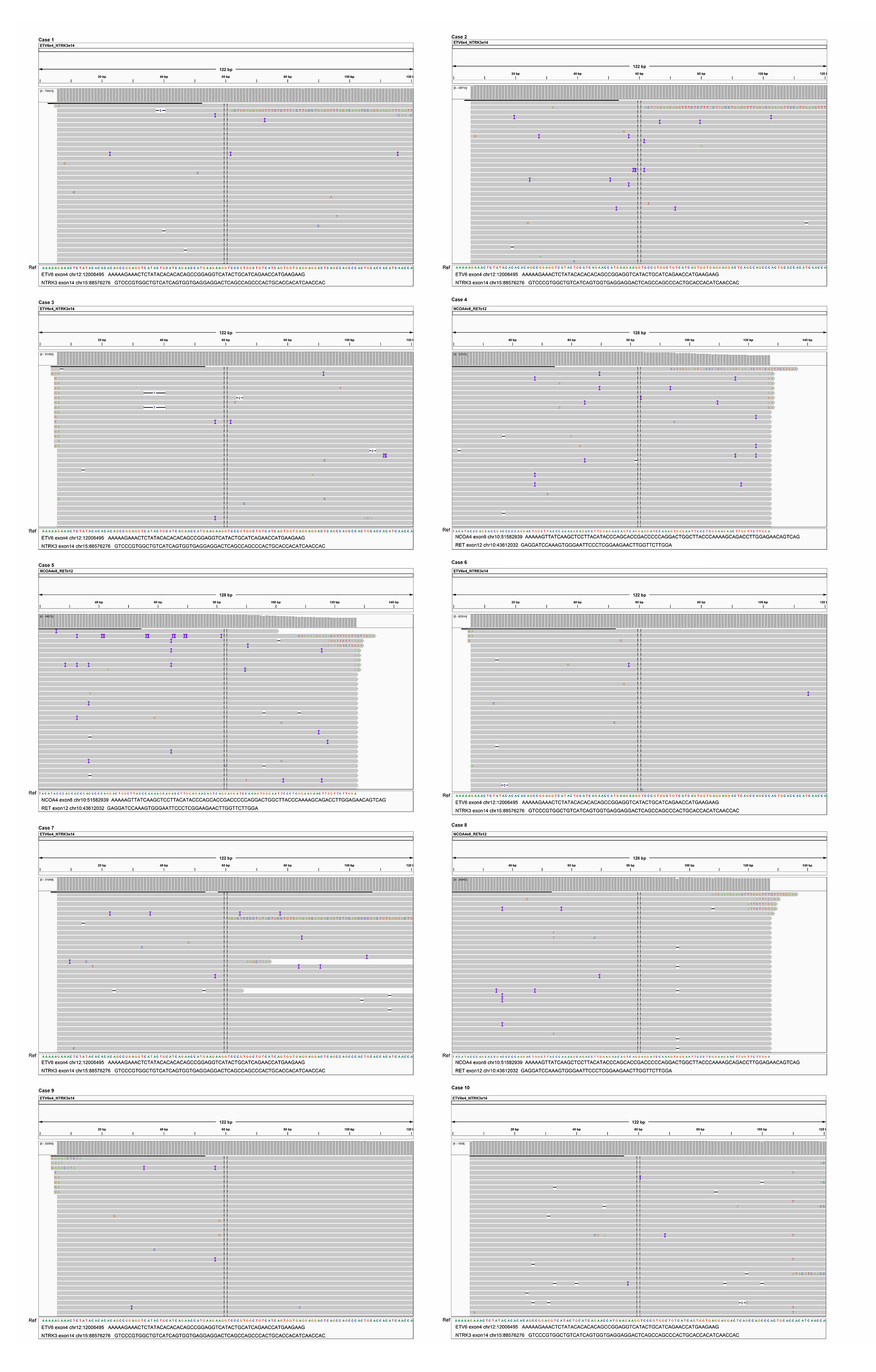

Supplement: Supplementary Figure 1 — The Integrative Genomics Viewer (IGV) screenshots display the reads from next-generation sequencing by Onco-Thyroid panel and reveal harbouring of gene fusions. The reference is fusion gene sequences. [file Image_1.tif]

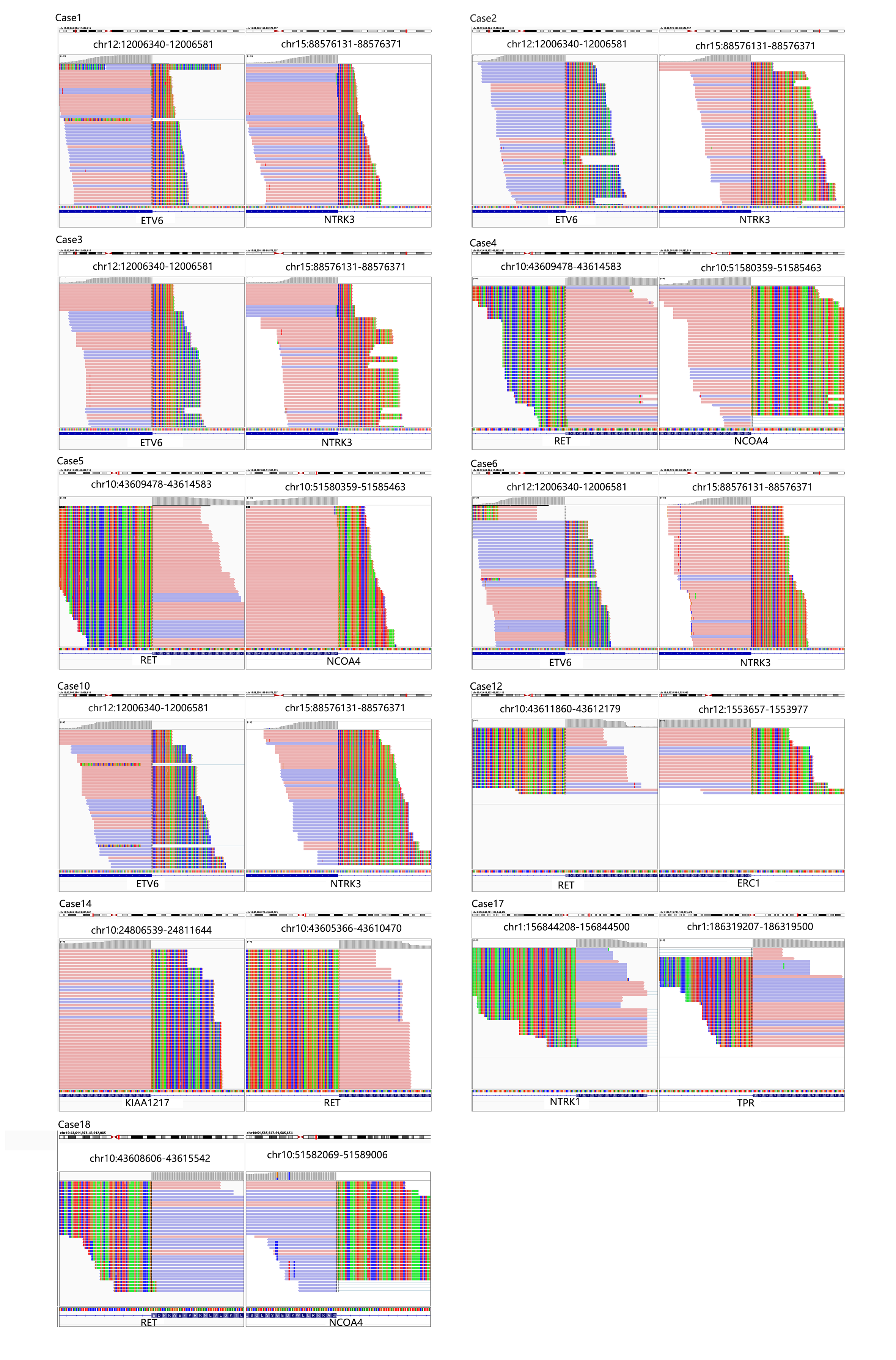

Supplement: Supplementary Figure 2 — The IGV screenshots display the reads from next-generation sequencing by Fusioncapture panel and reveal harbouring of gene fusions. The reference is GRCh37/hg19. [file Image_2.tif]

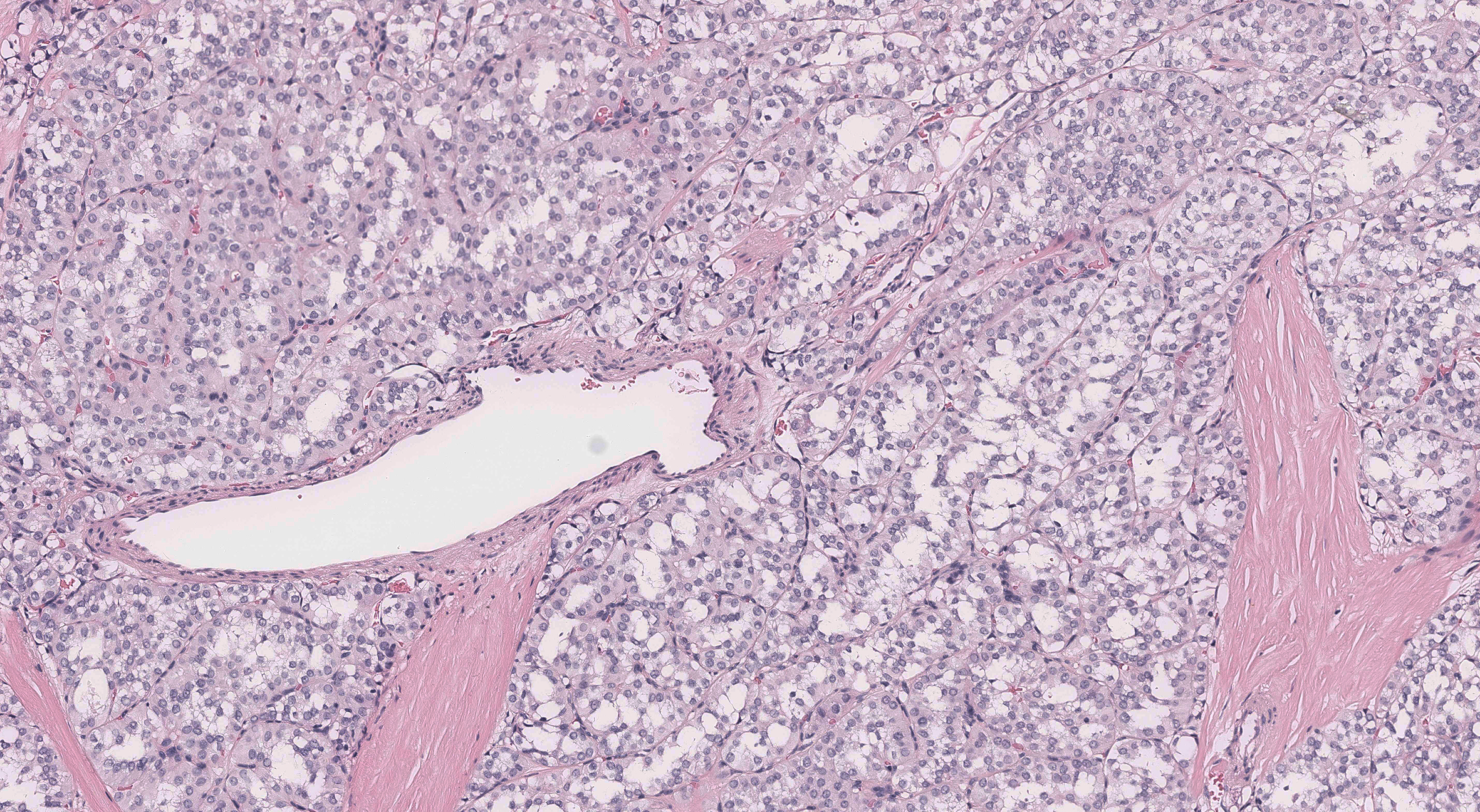

Supplement: Supplementary Figure 3 — The delicate papillae result from cellular dehiscence, with rugged surface and adhesion. [file Image_3.tif]

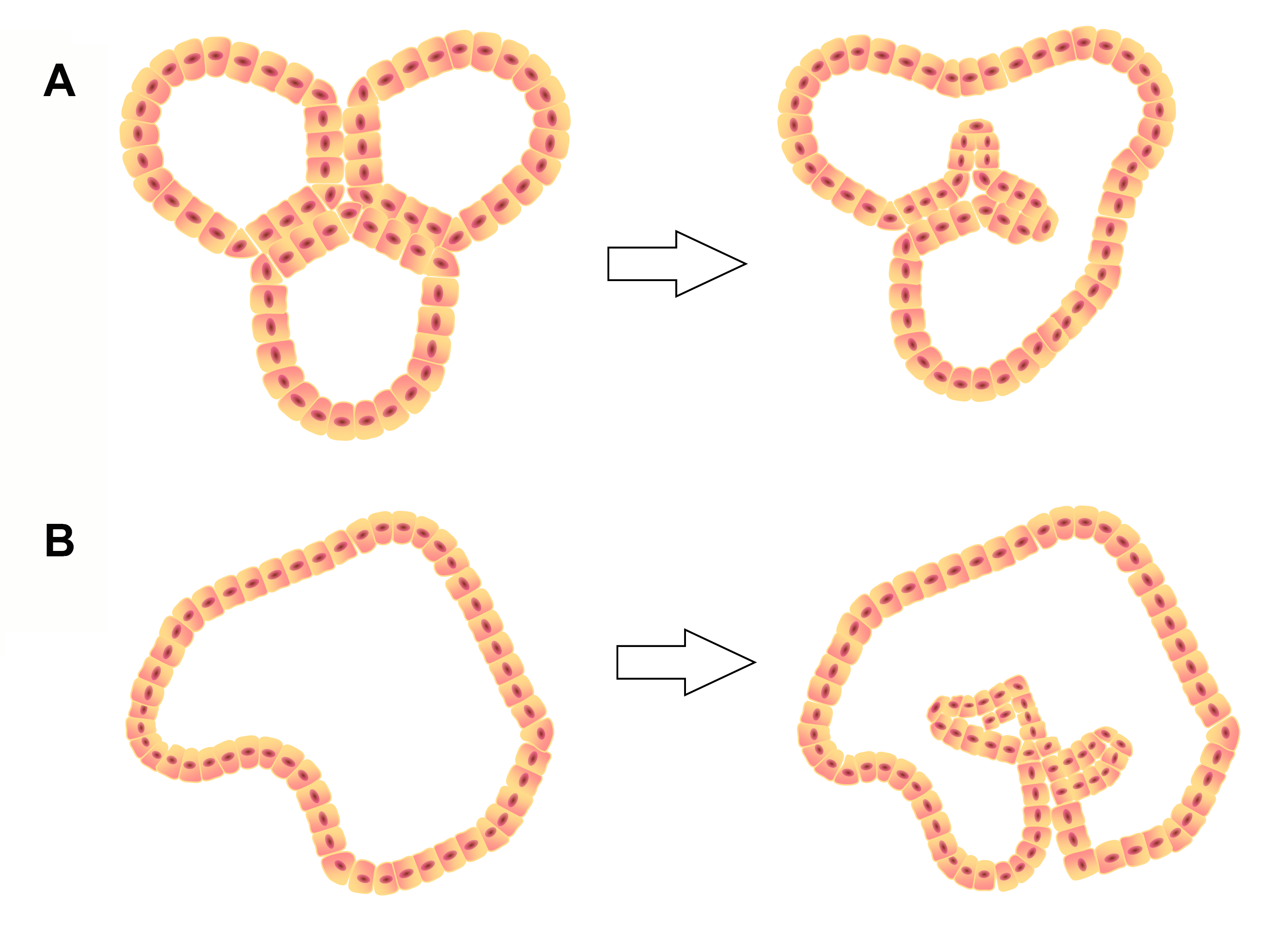

Supplement: Supplementary Figure 4 — Putative scheme of papillae formations. (A) The delicate papillae originate from packed microfollicles. (B) The common papillae originate from irregular follicles. [file Image_4.tif]
